# Epilepsy Successfully Treated by Bromide of Potassium

**Published:** 1865-10

**Authors:** 


### Epilepsy Successfully Treated by Bromide of Potassium.

M. Bazin has successfully treated a case of epilepsy by the bromide of pottassium. The patient was engaged in trade, forty years of age, of a sanguine temperament and robust constitution, but he had sunk into a melancholic condition; and one day he fell down in the public streets in an epileptic fit, which was succeeded by other attacks, recurring more and more frequently, until at last several occurred in the same day. In proportion as the attacks became more frequent the intellectual faculties were altered, as well as the functions of nutrition. Under these circumstances M. Bazin administered the bromide of pottassium in progressive doses and this treatment was immediately followed by a cessation of the fits; and in five months from the time when the treatment was commenced there had not been a single attack, and all the physical and intellectual functions were restored. In three other cases of epilepsy, in private practice, M. Bazin found the bromide of potassium equally successful, although the disease had previously resisted different kinds of treatment. The mode of administration adopted by M. Bazin was to form a solution of the bromide in the proportion of 20 grammes (a gramme is about 15 grains), to 300 grammes of distilled water; two tablespoonfuls of this solution, representing 30 grammes, contained 2 grammes of bromide. In the adult he commences at once with this quanity of two spoonfuls—one dose in the morning, and one in the evening, before meals. Every five days the dose is increased progressively by one spoonful up to eight or ten spoonfuls a day. This last dose is continued more or less, according to the degree of resistance of the disease. When the attacks are evidently modified, the quantity of the medicine is diminished in an inverse progression to four spoonfuls, a day, a quanity which is continued for several months, even after the cessation of the attacks. In children the medicine is given in the same manner, substituting a teaspoonful or less for the larger doses, according to the age of the patient.—*British & Foreign Med. Chir. Rev.*, July, 1865, from *Gazette des Hopitaux.*

